# Technology-Based Innovations in Child Maltreatment Prevention Programs: Examples from SafeCare®

**DOI:** 10.3390/socsci3030427

**Published:** 2014-08-15

**Authors:** Melissa Cowart-Osborne, Matthew Jackson, Elizabeth Chege, Evander Baker, Daniel Whitaker, Shannon Self-Brown

**Affiliations:** School of Public Health, Georgia State University, Atlanta, GA 30303, USA

**Keywords:** child maltreatment, SafeCare, child welfare, evidence-based programs, parenting, technology

## Abstract

Each year, hundreds of thousands of children in the U.S. are victims of child maltreatment. Experts recommend behavioral, skill-based parent training programs as a strategy for the prevention of child abuse and neglect. These programs can be enhanced using innovative technology strategies. This paper presents a brief history of the use of technology in SafeCare®, a home visiting program shown to prevent child neglect and physical abuse, and highlights current work that takes a technology-based hybrid approach to SafeCare delivery. With this unique approach, the provider brings a tablet computer to each session, and the parent interacts with the software to receive psychoeducation and modeling of target skills. The provider and parent then work together to practice the targeted skills until mastery is achieved. Initial findings from ongoing research of both of these strategies indicate that they show potential for improving engagement and use of positive parenting skills for parents and ease of implementation for providers. Future directions for technology enhancements in SafeCare are also presented.

## 1. Introduction

Child maltreatment (CM) is one of the most significant and substantive issues facing the U.S. today with a high incidence and at a staggering cost to the individual and society at large [1–3]. In 2012, an estimated 686,000 children were victims of abuse and neglect, an incidence rate of 9.2 per 1000 children in the population [4]. Over a quarter of these victims were less than three years of age, and approximately 20% were between the ages of 3 and 5. Over four-fifths of abuse and neglect cases (81.5%) were perpetrated by either one or both parents [4]. The incidence rates of CM are widely believed to be underestimates [1,3], and data show that the majority of CM incidents are not investigated by Child Protective Services (CPS) [5]. Although CPS reports indicate that approximately one percent of children in the U.S. are victims of CM each year [4], these data find that the percentage of children who are victims is closer to four percent [5].

Children who experience CM are at increased risk for poor emotional, social, and behavioral consequences [6] as well as increased risk for deleterious long term effects, including poor health and lower achievement, across the lifespan [7–9]. More specifically, adults who were CM victims have higher reports of poor lifestyle factors than adults who were not CM victims, as well as having higher incidences of smoking, severe obesity, mental illness, ischemic heart disease, cancer, chronic lung disease and liver disease [7]. Consequently, the projected lifetime economic burden of new victims of CM in 2008 alone was $124 billion. This figure, while staggering, is based on conservative assumptions, underscoring the importance and cost-effectiveness of CM prevention [2].

### 1.1. CM Prevention: Behavioral Parent Training Programs

According to data from the National Child Abuse and Neglect Data System (NCANDS), parents are the most common perpetrators of CM [4]. Thus, increased attention has been paid to prevention programs that target parents, especially those at risk. The risk factors believed to underlie CM include parental substance abuse, mental illness, domestic violence in the home (28.5% of victims) [4], child conduct problems, and poverty [1]. Interestingly, the interventions that have been the most successful in reducing CM are not those that address these risk factors but rather those that focus on improving parenting skills [1,10], known as Behavioral Parent Training (BPT) programs. Experts in the field recommend BPT programs as a type of evidence-based model that may benefit families in the child welfare system and serve as a CM prevention strategy [1,11,12].

BPT programs are based on the assumption that parenting skill deficits can be improved by providing parents with a repertoire of skills. They use a specific instructional format that includes: provider instruction and behavioral modeling of targeted skills, parent practice of skills through role plays and live practice with the child, and homework assignments for the parent, for example, to practice targeted interaction skills with the child outside [13]. The rationale for BPT programs in preventing CM is that parents who receive this training acquire and develop positive parenting skills and are less likely to use coercive methods to address challenging child behavior as well as experiencing changes in the attitudes that promote harsh parenting practices [8]. The evidence suggests that helping parents interact with their children and build parenting skills also benefits their mental health [1].

Research on several of these programs has shown that they can reduce the risk of CM perpetration and recidivism in a population of families at increased risk for maltreatment. For example, Chaffin and colleagues [11] conducted a randomized trial of families referred for physically abusive behavior comparing Parent–Child Interaction Therapy (PCIT) to a standard community-based parenting group and to PCIT with enhanced case-management services. Their findings indicate that 19% of PCIT-treated families had a re-report for physical abuse compared to 49% of families treated via the community-based parenting group. Additionally, in one of the few studies to examine the use of a BPT program as a primary prevention program, Prinz and colleagues [14] conducted a large population-level randomized trial of Triple P, another evidence-based BPT program, in South Carolina and results indicate a relative reduction in maltreatment rates for areas in which Triple P was implemented compared to areas where it was not.

More recently, several randomized trials have found the benefits of SafeCare as an effective program for reducing CM recidivism for parents of children 0–5 years. The program consists of three, six-session modules: Parent–Child Interaction (PCI), Child Health, and Home Safety. Each of these modules targets skills that are proximal to CM risk factors. In PCI, parents learn skills to increase positive interaction with their children and reduce challenging child behaviors. The home visitor teaches skills used to structure activities for children including daily routine activities, activities outside of the home, and independent play. Through the child health module, parents are taught how to respond to a variety of health-related situations that may arise. They learn to use health reference materials to help identify illnesses and symptoms and respond accordingly, whether it is by treating at home, making an appointment with the doctor, or going to the emergency room. The home safety module is intended to reduce the risk for unintentional injury by teaching parents to identify hazards in the home, to understand when objects are reachable or accessible by children, and how to remove hazards or make them inaccessible. Parents also learn the importance of appropriate supervision. The first and last sessions in each module are assessment sessions in which the home visitor assesses the parent's skills. In this way, the home visitor can track the parent's skill acquisition. Sessions two through five are training sessions in which the parent learns the material for that session. Each training session is structured around four components: Explain, Model, Practice, and Feedback. As this description implies, the home visitor begins by explaining the skills, then models the skills for the parent, allows the parent to practice the skills, and, finally, the home visitor provides positive and corrective feedback to the parent regarding their use of the skills [15].

SafeCare is a unique BPT program in that it is delivered in the home setting as opposed to a clinic. This delivery strategy provides the benefits of reducing logistical barriers for families, while also offering service providers an increased opportunity to understand factors occurring in the ecological context of the home environment that influence parent decision making and behavior. By delivering the curriculum in this manner, the provider is able to directly observe parent's use of the skills in the home, where they will most often be implemented, and also provide feedback during training in the home environment [16]. Additionally, SafeCare was designed specifically to prevent CM as the primary program objective. The three SafeCare modules address proximal risk factors for both child physical abuse as well as neglect, the most prevalent form of CM [4]. Other BPT programs were developed to target different outcomes and have been applied to CM prevention following positive results for proxy behavioral outcomes.

The largest study to date is a statewide comparative effectiveness trial of SafeCare in the Oklahoma child welfare system. Chaffin and colleagues [17] conducted a six-year longitudinal study comparing rates of recidivism between a group of parents receiving SafeCare services and a control group receiving family preservation services as usual (n = 2175). This study found that SafeCare prevented 26% of repeat cases of maltreatment compared to the control group. Participation in SafeCare was also shown to improve parenting behaviors. Separate analyses found that SafeCare was equally effective for a large sample of American Indians who participated in the trial compared to non-minority participants [18]. A second randomized trial by Silovsky and colleagues [19] found differences on a range of outcomes (parent social support, child abuse potential, parent depression) favoring SafeCare as compared to usual services. A third trial also showed results favoring SafeCare participation over services as usual [20]. This study found that SafeCare parents had a higher service retention rate, greater use of non-violent discipline, and fewer child welfare reports related to domestic violence compared to the group of parents receiving services as usual. In addition to these randomized controlled trials, quasi-experimental and single-subject research has been conducted on SafeCare yielding similar results, all supporting the positive effects of SafeCare [21–24].

### 1.2. Using Technology to Enhance BPTs

The overall public health impact of a program includes the effectiveness of the intervention on targeted outcomes, as well as the reach or the percent and representativeness of individuals willing to participate [25]. While there is increasing evidence for BPT programs to reduce CM risk and incidence for families who complete, there are still significant limitations to the reach of these programs. Family service engagement, defined as parent beliefs about treatment that impact service participation and satisfaction, alliance with interventionist, and, ultimately, service retention [26], is one of the major challenges documented by research [27,28]. Emerging research indicates that, by increasing communication between provider and client and providing information in a unique way, technology-assisted evidence-based BPT programs delivered in clinics [29] and homes [30,31] can improve parent engagement and retention and, consequently, targeted outcomes.

In a special issue of the journal *Child Maltreatment*, Guest Editors Self-Brown and Whitaker [32] called for an increase in the use of technology for intervention delivery to parents to improve reach of evidence-based programs in the CM field. The growing penetration of internet and smartphone usage coupled with the growing resource constraints on state support of families at high risk for CM presents a great opportunity to promote innovative technology strategies. In addition to improving reach, these strategies afford the opportunity to reduce the costs of BPT provision while enhancing home visitor fidelity, or adherence to the intervention protocol [3]. Technology-enhanced BPT programs have the advantage of being adaptable to the individual and overcoming the challenges of implementation fidelity which arise from the incomplete adherence of interventionists to intervention protocols [6]. Delivery of the program through a computer ensures that the information is being delivered as intended in the intervention protocol (*i.e*., with fidelity). Also, in situations in which providers are delivering an evidence-based practice for the first time and are unaccustomed to following a structured protocol and experiencing increased supervision, the addition of a technology component could alleviate some of the stress related to a new implementation. Numerous research studies have shown that technology can successfully aide in delivery of these programs. What follows is a discussion of how technology has been implemented in SafeCare in the efforts to prevent child abuse and neglect.

## 2. Literature Review of the Use of Technology in a BPT Program: A Look at Previous SafeCare Strategies

The introduction of technology into SafeCare first emerged in the mid-1980s when home visitors successfully utilized an audio slide show to help parents identify safety hazards [33]. Over a decade later, video training was tested in the PCI and home safety modules. In this approach, home visitors brought a ten-minute video, the content of which covered the explaining and modeling portions of that session, to each visit. The video was played on the parent's VCR while the home visitor monitored the child. A single subject study of two parents was conducted for the PCI module and for the home safety module. Results of these studies found that parents’ skills improved greatly after the video training was implemented and that parents positively rated the video content and the use of video as an intervention delivery mechanism [21,34].

Since that time technology has improved and expanded greatly, and SafeCare has adapted to incorporate newer technologies. More recent technological augmentation of SafeCare has included use of the iPhone™ in both service delivery and data collection. Jabaley and colleagues [35] utilized a technology-assisted approach to the delivery of the home safety module, such that in addition to the standard home visits, an iPhone was given to parents as a way for parents to capture video of rooms in their home, which they sent to their home visitor in between sessions. The home visitor was then able to remotely assess the room for hazards and provide the parent with praise and feedback. The phone also functioned as a way for the home visitor to communicate with the parent between sessions, providing tips and feedback and reminding the parent of upcoming sessions. In this way, the technology served not only to aide in service delivery and data collection but also as a way to increase engagement. This technology-assisted version of the SafeCare home safety module successfully reduced home hazards across 3 at-risk families and the intervention module was delivered fully in five sessions as opposed to six sessions with the assistance of the technology.

Another way that technology was used to enhance SafeCare is through the use of a digital frame as an enhancement to the Parent–Infant Interaction module. In SafeCare sessions, the home visitor took pictures of the parent as she correctly modeled the skills and uploaded them to the digital frame. The home visitor and mother then reviewed the pictures together to ensure that the mother knew what skills she was using in each picture. The frame was then set up in the home with the pictures shown on a continuous loop. The home visitor helped the mother schedule times when she could practice the skills, using the digital frame as a guide during practice. A single subject study of this approach was conducted with a mother with intellectual disabilities in which the mother increased substantially in her use of both physical (83%) and non-physical (50%–62.5%) parent-infant interaction skills upon completion of training. This increase was sustained at a three-month follow-up [36]. As with the case of the iPhone, technology enhanced the program in this example by providing the parent with a review of the skills between sessions.

Carta and colleagues [30] found success with a technology-enhanced SafeCare adaptation in a randomized controlled trial (n = 371 mother-child dyads). In this study they tested the efficacy of adding a cellular phone component to Planned Activities Training (PAT), a key component of the SafeCare PCI module. The mother-child dyads were randomly assigned to one of three conditions: (1) PAT (the SafeCare PCI module); (2) CPAT (the PCI module plus cell phone assistance); or (3) Waitlist Control. Mothers assigned to the PAT group participated in standard SafeCare PCI in-home sessions with a trained home visitor. The CPAT group received a technologically-augmented intervention that involved simple cell phone text messages and phone calls from the home visitor in addition to the PAT sessions, increasing the contact between home visitors and mothers. Specifically, home visitors would send out texts twice a day. One text was used to remind the mother of a certain PAT skill and one contained a question related to her skills usage or the child's behavior. These messages were tailored to the specific mother's issues that came about during the in-person sessions. Home visitors also called the mothers once a week in order to discuss the content of the text messages as well anything else the mothers felt warranted discussion; these talks were directed by the mothers.

Results showed that both the parents in the PAT and in the CPAT groups utilized significantly more positive parenting skills than the wait-list control group, and children of these mothers had more positive engagement compared to the control group. Furthermore, the CPAT group demonstrated even higher positive parenting strategies in addition to reduced parenting stress and depression and increased child adaptive behaviors compared to the other two groups. Additionally, data suggested that parents in the cell phone group had greater retention in services [31].

These examples highlight ways that technology has been employed to enhance SafeCare in the past. This paper presents two ongoing studies that integrate technology in a hybrid approach to delivering SafeCare. The first will examine family-level data in a technology-enhanced program designed specifically for fathers. The second study will assess outcomes at the provider level in a technology-assisted home visiting program for parents of young children.

## 3. Current Work Examining Technology-Based Enhancements in SafeCare

An innovative approach to technology in parenting programs targeting home visiting is to consider how program providers can utilize technology devices during service delivery to advance client engagement and associated retention. Increasingly, service providers who work with families in the home are provided laptops, smartphones, or tablets by their employers [37], which could simply be utilized in session to offer parents access to web-based information. In recent years, federal grant funding from the National Institutes of Health has allowed for the development of a web-based SafeCare program that can be used during home visiting sessions. The web program was designed using best principles of communication theory, with personally and contextually tailored health information, user-centered design and interactivity, as well as the use of multimodal teaching that can enhance individual engagement, learning, and potential behavior change [38,39]. SafeCare providers take a technology-assisted, computer-mediated approach [40] to session delivery in which the computer is used as a third partner in the relationship between a provider and client. That is, the home visitor connects the parent to the web-based program, and the parent participates in the multimodal learning (e.g., explanation and modeling of skills) of SafeCare target skills. When the parent completes the web-directed portion of the session, the provider takes over and has the parent engage in live practice of the skills discussed in the web program and provides positive and constructive feedback. This approach offers the advantages of computer intervention delivery, without eliminating the provider-client relationship that can be key to child safety when working with at-risk families. Two studies are currently utilizing this technique in SafeCare, Dad2K and SafeCare Takes Care. Both of these SafeCare augmentations are being explored through randomized controlled trials, which have been approved by the Georgia State University Institutional Review Board. These studies and early findings are described below.

### 3.1. Dad2K: Dads to Kids

#### Description of Dad2K

Dad2K is a technology-assisted delivery approach to SafeCare PCI that has been adapted for fathers. The sessions are action-oriented, and even fathers who have limited visitation with their children will find the interaction skills they learn in this module applicable. In addition to the possibility of fathers having limited visitation with their children, they may also have transient living arrangements, making home visits a challenge. In the SafeCare PCI module, home visitors can conduct the sessions virtually anywhere. For instance, if a father is living with a family member or has visitation in a public setting such as a park, the home visitor can easily conduct the session at those locations. These features make SafeCare PCI especially relevant as the foundation for a program for fathers.

Development of the program was an iterative process, taking place over the course of approximately nine months. Modifications were made to the program to make it more appealing to men. For instance, a sports theme was integrated throughout the program, and the program was named Dad2K, which is a play on the title of a popular sports video game. Also, in PCI, parents complete an “Assessment Form”. In Dad2K, the same form is used, but it is referred to as a “Scorecard”. Videos in the computer portion of the sessions are hosted by a male “coach”, who explains the skills using sports analogies throughout the program; the modeling in the videos is conducted by a father.

Co-parenting is a domain not formally addressed in SafeCare that is dealt with in Dad2K. This component was added to the program due to the possibility that fathers may have custody issues that present unique challenges in co-parenting. At the end of session four, the home visitor asks the father about any conflicts he has with his co-parent related to child rearing. Using this situation or an example of one, the home visitor completes the SafeCare Problem Solving Worksheet with the father, asking him to list all of the possible options to address the problem and the pros and cons of each.

Finally, a technology component was added to the program, creating a hybrid approach to home visiting, as described above. At the beginning of each session, the parent completes the Explain and Model portions of the session via the online computer program. The program is accessed via a tablet and Internet hotspot that the home visitor brings to each session. The home visitor then resumes with the Practice and Feedback portions of the session. The hybrid approach to delivery may be especially relevant to fathers, as previous research has found that fathers do not respond as favorably to the typical structure of parenting programs (*i.e.*, one-on-one discussion with a therapist or home visitor) as mothers do [41]. This dynamic approach to the session structure, beginning with an interactive computer portion and moving to a more traditional method of parent training, may increase their engagement and interest.

#### Dad2K Home Visitor Training

Two female SafeCare Training Specialists and one male PhD student, who has experience delivering father programs, were trained to deliver Dad2K. All three were trained to deliver SafeCare. In addition to SafeCare training, which consists of didactic training as well as field work, the home visitors took part in additional training to familiarize them with the adaptations made to PCI in the Dad2K program, as well as to teach them how to use conduct the computer portion of the session.

#### Dad2K Efficacy Trial and Initial Experience

Funded by the National Institute of Health and Health Disparities, 120 fathers between ages 18–30 with at least one child between the ages of 2–5 years are being recruited from community organizations, local businesses, and neighborhoods in the Atlanta area for the randomized controlled trial to test Dad2K against a control group that receives parenting materials by mail. Thus far, fourteen fathers have enrolled in the study. They are all African American with a mean age of 25.1 years. Fifty-seven percent report an annual income of less than $15,000, and 43% have a high school diploma or General Educational Development (GED) exam (*i.e.*, high school equivalency) or less.

To date, the rate of program completion for the intervention group has exceeded expectations and typical completion rates found in the literature [27,28]. Of the seven participants in the Dad2K group, four have completed the program and two are actively participating. One participant has been lost to attrition. Participants (n = 4) have rated their satisfaction with the program as high. On a satisfaction survey completed at the end of the final Dad2K session, fathers were asked to rate items on a scale from one (“Strongly Agree”) to five (“Strongly Disagree”). The mean rating for the item, “Interacting with my child has become easier”, was 1.5, with 1, “Strongly Agree”, being the most favorable rating. With regard to “I have more ideas about activities I would like to do with my child”, and “Routine activities...have become easier”, three out of the four participants rated these items a one; the fourth rated it a two. The mean participant rating for the item, “I liked doing part of my session on the computer”, was 2.00.

Dad2K home visitors have reported positive feedback from the fathers participating in the intervention. They note that fathers enjoy discussing parenting issues and the sessions sometimes last longer than average SafeCare PCI sessions due to fathers’ questions and engaging discussion. Thus far, there have only been two instances of the Dad2K computer portion of the session not being completed due to technical problems. After pilot testing, a protocol was developed whereby home visitors make two attempts at troubleshooting computer problems before they revert to the traditional home visiting method of delivery. If the technical problem is due to Internet connectivity issues, the home visitor can still show the videos to the father, as they are saved directly on the tablet.

The efficacy trial is expected to be completed by August 2016. At that time, the research team will be able to examine how effective Dad2K was in improving maltreatment risk, father–child interaction, father mental health, child behavior, and father involvement.

### 3.2. SafeCare Takes Care Program

#### Description of SafeCare Takes Care (SC-TC)

SC-TC also involves augmentations to SafeCare. However, in this case, the modifications are primarily concerned with the addition of technology to the program rather than use of the program in an atypical group of participants, and the program includes all three modules instead of just PCI. Just as with Dad2K, the home visitor brings a tablet and Internet hotspot, and the session begins with the parent viewing the Explaining and Modeling components through the computer program. The computer portion of SC-TC also involves a combination of video, audio narration, and engaging questions. However, the videos in this program have a talk show theme rather than a sports theme. For each module and session, the host of “SafeCare Takes Care” presents a new topic (*i.e.*, the session content for that day) with video modeling of the skills from “at-home viewers”. For example, in PCI, a video begins with the talk show host explaining the skills being covered in the session, then the parent will see video of a parent modeling these skills, and the host may take some questions from studio audience members or from fans on the “street cam”. Following completion of the computer portion of the session, the parent practices the skills and receives feedback from the home visitor.

#### SC-TC Home Visitor Training

All home visitors in the SC-TC study receive SafeCare training as usual. This consists of four days of classroom training that involves didactic presentations, modeling of the instruction, and role playing. Home visitors assigned to the SC-TC group receive an additional half day of classroom training in which they go through the computer program on a tablet, learning how to navigate the tablet and the computer program. Following classroom training home visitors in both the SC-TC group and the SafeCare as usual group conduct field work in order to reach certification. After certification, home visitors continue to be fidelity monitored and coached on a monthly basis.

#### SC-TC Randomized Trial and Initial Experience

Funded by the National Institute of Mental Health, the study of SC-TC chiefly examines how well the technology-assisted approach affects implementation outcomes for providers, as well as some basic family-level data. Thirty SafeCare home visitors will be recruited for the study and will either be assigned to standard SafeCare training, or the technology-assisted training (SafeCare Takes Care group). Each provider participant will be asked to recruit two families, resulting in 60 SafeCare families in the sample. This study is in the very early stages of data collection and currently has three home visitor participants enrolled. Two SafeCare parents have been enrolled in the study. One of the home visitors has a graduate degree, and the remaining two have Bachelor degrees. They have all been working in the field for at least six years with two of the participants having ten or more years of experience. Both SafeCare parents are Caucasian females with a mean age of 33. They both report an income of less than $20,000 annually.

Informal, qualitative data obtained from home visitors in the SC-TC group suggest that they find the program feasible and even preferable to standard delivery. Specifically, they have found that the videos and content in the computer portion sparks discussion with the parents, strengthening the session topics. To date, there have been no technological malfunctions precluding session completion in SC-TC. The study will be completed in April 2015, and upon completion the research team will examine whether the technology-assisted providers attain SafeCare certification, maintain fidelity, and sustain delivering SafeCare services at higher rates that providers trained in SafeCare as usual. Family-level outcomes such as maltreatment risk and child behavior will also be explored.

## 4. Conclusions

This paper discussed the use of technology in a CM prevention program and the promise this approach shows in engaging parents, improving the use of positive parenting skills, and easing implementation for providers. Although few conclusions can be drawn at this point in the research process based on the current number of participants enrolled in these studies, the early findings indicate that addition of technology to the program is beneficial to home visitors and parents. In addition to CM prevention, technology enhancement can positively affect outcomes in other programs targeted toward parents. Recent work has illustrated how technology is also being introduced into programs that aim to improve child outcomes by addressing infant social development and child externalizing behaviors. For instance, Baggett and colleagues [6] developed a completely Internet-based adaptation of an evidence-based parenting program that has been found to improve infant developmental outcomes. Their research found that there was a high retention rate, high satisfaction and ease of use, and improved parent and child outcomes compared to a control group. In a study of a program targeting parents of children with disruptive behavior [29], a technology-enhanced version of the program included addition of videos, text message reminders, video recording home practice, and midweek video calls. This study showed that the technology-enhanced group had significant decreases in disruptive behavior, increases in customer satisfaction, and lower implementation costs than the group receiving the standard protocol. It is evident that technology can be used in multiple approaches to enhance, not only CM prevention programs, but programs that address other infant and child outcomes as well.

Technology is rapidly growing and changing, and it is increasingly permeating daily life. Thus, in order for CM prevention programs to stay relevant and engaging to a broad population, technology-based approaches must adapt to these changes. This is especially true in considering work with adolescent parents who are accustomed to both multimodal learning and communication through technology and the Internet, as this has been available for their entire lives.

Also important to consider is access to technology and to the Internet. Internet usage by adults in the U.S. increased overall from 79% in 2010 to 87% in 2014, and smart phone usage has increased from just 35% in 2011 to 58% in 2014 [42]. Despite these increases in overall usage, a digital divide remains. When examining Internet and smart phone use by socioeconomic factors such as education or income, there are significant differences in access. Essentially, there is still a full 20% of Americans who have neither access to a home Internet connection nor to a smartphone [43]. For families without access to technology at home, programs such as Dad2K and SC-TC, in which providers bring the technology to the sessions, will allow them to receive the benefits that technology offers without expecting that the families will supply it. Government leadership has recognized that access to technology is important, and White House initiatives are planned to expand Internet access to at least 98% of Americans [44]. As time goes on and access increases and costs decrease, CM prevention programs should incorporate technology enhancements as part of standard protocol, and future research should examine the feasibility of implementing a technology-enhanced program using parents’ own technology.

With the need to adapt to expanding technology in mind, new approaches to technology-based enhancements in the SafeCare program are under way. One example of a technology enhancement that is already being utilized by SafeCare home visitors and trainers is the SafeCare Portal. This is a secure website to which home visitors upload audio files of their SafeCare sessions for their trainer to listen to for continued fidelity monitoring and where they can track their progress toward certification as a SafeCare home visitor. Before development of the Portal, home visitors uploaded audios to a secure third-party website in a process that could sometimes be confusing for home visitors and for SafeCare trainers. The Portal has made this process simpler, which can ease home visitor job demands, especially for those sites new to implementing an evidence-based practice that requires fidelity monitoring. In addition to the SafeCare Portal, researchers at the National SafeCare Training and Research Center are developing new mobile tools through funding from the Agency for Healthcare Research and Quality. These tools will allow home visitors to collect data on parents’ skill acquisition as they participate in SafeCare, track parent engagement and alert the home visitor if engagement is low, and upload a home visitor's session audio files directly to the SafeCare Portal. These enhancements have the potential to increase retention, allow home visitors to tailor each parent's sessions based on their needs and level of engagement, and to ease the implementation burden on home visitors. Technology is a significant and innovative approach that can be used to strengthen the public health impact of CM prevention programs by increasing reach, relevance, and engagement for families who are at greatest risk.
